# Would Illness Representations of COVID-19 and COVID-19 Fears During Clinic Visits Promote or Reduce Behavioral Intention to Seek Medical Consultations for Flu Symptoms? A Random Telephone Survey in Hong Kong, China

**DOI:** 10.3389/fpubh.2022.903290

**Published:** 2022-06-10

**Authors:** Sitong Luo, Rui She, Mason M. C. Lau, Joseph T. F. Lau

**Affiliations:** ^1^Vanke School of Public Health, Tsinghua University, Beijing, China; ^2^Institute for Healthy China, Tsinghua University, Beijing, China; ^3^Centre for Health Behaviors Research, Jockey Club School of Public Health and Primary Care, The Chinese University of Hong Kong, Shatin, Hong Kong SAR, China

**Keywords:** coronavirus disease 2019, flu symptoms, medical consultation, behavioral intention, illness representations, fear

## Abstract

**Background:**

The study investigated the level of behavioral intention to consult doctors for flu symptoms (BICDFS) during the Coronavirus Disease 2019 (COVID-19) pandemic and examined its associations with illness representations of COVID-19 and fear of COVID-19 during clinic visits in a general Chinese adult population.

**Methods:**

A random telephone survey was conducted among 300 residents in Hong Kong, China in April 2020 when the second wave of COVID-19 was just ended in the region. The participants were asked about their intention to consult doctors if they had mild or severe flu symptoms in the next week (from 1 = *definitely no to 5* = *definitely yes*). Illness representations of COVID-19 were measured by the Chinese version of the Brief Illness Perception Questionnaire (BIPQ). The fear of COVID-19 during clinic visits were assessed by two single items. Linear regression adjusted for background variables and hierarchical strategies were employed.

**Results:**

Of the participants, 52.3 and 92.0% showed an intention to consult doctors for mild and severe flu symptoms, respectively. Adjusted for background factors, COVID-19-related cognitive representations (consequences: *standardized b* = 0.15, *p* = 0.010; understanding: *standardized b* = 0.21, *p* = 0.001) and emotional representations (concern: *standardized b* = 0.17, *p* = 0.001; negative emotions: *standardized b* = 0.19, *p* = 0.001) were positively associated with BICDFS. In the hierarchical model, independent significant factors of BICDFS included understanding (*standardized b* = 0.16, *p* = 0.013) and negative emotions (*standardized b* = 0.17, *p* = 0.008). The fear-related variables showed non-significant associations with the BICDFS.

**Conclusions:**

Promotion of care-seeking behaviors for flu symptoms during the COVID-19 pandemic should consider improving people's understanding of COVID-19 and providing advice on related coping strategies for emotional responses to COVID-19.

## Background

As of 11 March 2022, the Coronavirus Disease 2019 (COVID-19) had caused over 433 million infections and over 5.9 million deaths worldwide (1, 2), and over 570,000 infections and 3,150 deaths in Hong Kong, China where the study was conducted (3). The COVID-19 pandemic is still going on around the world, and the disease is likely to become a seasonal illness (4).

During public health emergencies, people's care-seeking behaviors may be dramatically affected (5). For example, at the peak of the SARS epidemic, Taiwan observed a significant reduction in the use of ambulatory care, inpatient care, and dental care (6). During the Ebola outbreak, the utilization of inpatient and outpatient services declined by 18% on average in Western Africa (7). As COVID-19 shares some similar symptoms with flu, such as fever, cough, tiredness, and sore throat (8), the miss of timely medical consultation for flu-like symptoms may result in under-diagnosis of COVID-19 and other diseases with similar clinical features, such as influenza, tuberculosis, and malaria (9). Few studies had evaluated the general public's utilization of medical consultations for flu symptoms during the COVID-19 pandemic.

Illness representation, which is the beliefs and perceptions about an illness, may influence people's care-seeking behaviors (10, 11). According to the Common Sense Model of Self-Regulation, a health threat stimulus, can activate two sets of illness representations: cognitive representations for regulating the objective health threat and emotional representations for appraising and managing their emotions (12). The two sets of illness representations are constructed based on one's available information about the health threat, and then guide people to either positively or negatively cope with the threat (12). For instance, a previous study on H1N1 in Hong Kong showed that the perception of better controllability of H1N1 treatment and the perception of less psychological attribution of H1N1 were significantly associated with a higher intention of receiving influenza vaccination, while emotional representations (i.e., negative emotions aroused by H1N1) were correlated with a lower frequency of face-mask wearing and handwashing (10). Another study in Greece reported that emotional representations of H1N1 were associated with a higher frequency of primary and secondary preventive behaviors (13). It is hence expected that various dimensions of illness representations on COVID-19 may also trigger either positive coping (e.g., consulting doctors for related symptoms) or negative coping (e.g., avoiding care-seeking) with the disease. Few studies had explored such relationships.

Fear is another common factor affecting people's care-seeking behaviors in disease outbreaks (6, 14–16). For example, fear of SARS was found to disproportionally reduce people's utilization of medical services (6) and cause undetected SARS cases in the community (14). Fear of Ebola impeded the care-seeking behaviors of both Ebola patients and patients with other serious medical conditions (16). According to the fear appeal theory, a health threat can arouse people's fear and then initiate a dominant response of danger control which may prompt people's protective actions (17, 18). As hospital-acquired infection (HAI) occurred frequently in previous infectious disease outbreaks (e.g., SARS) (19), people may tend to see health facilities as the riskiest place of infection and choose not to go there during the outbreak. During the COVID-19 pandemic, COVID-19-related fears may either motivate or hinder people's utilization of medical consultation for flu symptoms. On one hand, as COVID-19 infections could be HAI (20, 21) and visiting health facilities physically is the predominant way for medical consultations in many places (e.g., Hong Kong), fear of COVID-19 may thus drive people not to visit hospitals/clinics to avoid cross-infection. On the other hand, as flu symptoms are clinical features of COVID-19, fear of COVID-19 may urge people to seek medical help promptly to confirm diagnosis and receive treatment. Moreover, postulated by the extended parallel process model, in addition to the danger control response, a health threat can also invoke a fear control response (i.e., controlling their fear instead of controlling the disease), which potentially causes avoided/delayed care-seeking behaviors for the threat (22). To the best of our knowledge, few studies had investigated the association between COVID-19-related fears (e.g., fear of COVID-19 during clinic visits) and people's medical consultation seeking behaviors for flu symptoms.

As the behavioral intention is an immediate antecedent of an actual behavior (23), this study measured the behavioral intention to consult doctors for flu symptoms (BICDFS). The aims of this study included: (1) to investigate the level of BICDFS during the COVID-19 pandemic in the general adult population in Hong Kong, China; (2) to examine the associations of BICDFS with each dimension of the illness representations on COVID-19 and each of the two types of fear of COVID-19 during clinic visits, and (3) to explore whether fear of COVID-19 during clinic visits would explain a significant amount of variance in the BICDFS after accounting for illness representations of COVID-19.

## Methods

### Study Design

This study was a random telephone survey conducted during 21–28 April 2020 in Hong Kong, China when the second wave of COVID-19 was just ended in the region. Eligible criteria included: (1) being ≥18 years old; (2) being able to communicate in Chinese (Cantonese or Mandarin); and (3) having a Hong Kong ID card. The trained interviewers randomly selected and called the household telephone numbers from the most up-to-date telephone directories in Hong Kong. To avoid under-sampling of employed individuals, telephone calls were made between 6:00–10:00 pm on weekdays and 2:00–9:00 pm on Saturdays. If no one in the household answered the initial call, up to four more follow-up calls would be made at different hours and days before it was considered as a non-valid telephone number.

At the beginning of the telephone interview, a screening question was asked to identify households with eligible participants. If a household had more than one potential participant, the one whose birthday was the closest to the interview date was invited to join the survey. Guarantees were made on anonymity and their right to quit at any time. Verbal instead of written informed consent was obtained prior to the interview. The interviewers signed a form pledging that the participants had been fully informed about the study. A total of 552 participants were invited to join the study; 300 provided verbal informed consent and completed the interview (response rate: 54.3%). No incentive was given to the participants.

### Measures

#### Background Characteristics

Sex, age, marital status, education level, and whether having had chronic diseases notified by doctors (e.g., hypertension, diabetes, cancer) were collected.

#### Behavioral Intention to Consult Doctors for Flu Symptoms

The study used two items to measure the level of BICDFS: whether you would consult doctors in the next week if you had: (1) mild; and (2) severe flu symptoms. The items were adapted from previous studies that investigated health-seeking behaviors during the SARS and H1N1 outbreaks in Hong Kong, China (10, 24). The response categories included 1 = *definitely no*, 2 = *probably no*, 3 = *probably yes*, and 4 = *definitely yes*. Those who answered *probably/definitely yes* were categorized as having an intention to consult doctors for corresponding symptoms. The score of the two items was summed up to construct a BICDFS scale, with a higher score indicating a higher level of BICDFS (range: 2–8). The Cronbach's alpha of the scale was 0.61, which was acceptable due the limited number of items in the scale (25). The Spearman correlation coefficient of the two items was 0.51 (*p* < 0.001), yielding a moderate strength relationship.

#### Illness Perception of COVID-19

The eight items of the Brief Illness Perception Questionnaire (BIPQ) were used to measure the participants' illness representations on COVID-19 (26). Each item represented a dimension of the illness representations. The cognitive illness representations were assessed by five items (consequences, timeline, personal control, treatment control, and identity); the emotional representations were evaluated by two items (concern and emotions); the other item measured the illness comprehensibility (understanding). Each item was rated on a 10-point scale. A higher score in dimensions of consequences, timeline, identity, concern, and emotions indicated the representations to be more negative, while a higher score in the dimensions of personal control, treatment control, and understanding indicated the representations to be more positive. The BIPQ has been previously validated in the Chinese context and showed good reliability and validity (27).

#### Fear of COVID-19 During Clinic Visits

The study used two items to assess the level of fear of COVID-19 during clinic visits: (1) fear of contracting COVID-19 in clinics if you would visit there to consult doctors in the next week; and (2) fear of being requested to take up COVID-19 testing if you would visit clinics for flu symptoms in the next week. The items were also adapted from previous studies on the SARS and H1N1 outbreaks in Hong Kong (28, 29). The response categories of each item were 1 = *not afraid at all*, 2 = *not afraid*, 3 = *neutra*l, 4 = *afraid*, 5 = *very afraid*, with a higher score indicating a higher level of the fear.

### Statistical Analyses

Distributions of all studied variables were described by frequencies and proportions or means and standard deviations (SD). Simple linear regression was used to identify background variables associated with the level of BICDFS. The significant background variables (*p* < 0.05) were controlled in the subsequent regression analyses on the associations between BIPQ items/fear items and the level of BICDFS. The Spearman correlation coefficients were derived to explore the crude associations between the BIPQ items, fear-related items, and BICDFS, as they were not normally distributed. A series of linear regression models were performed to examine the adjusted associations of BICDFS with each BIPQ item and each fear-related item after controlling for the significant background variables, which was because that each item represented a dimension of illness representations or fears.

Hierarchical regression analysis was then performed to test the improvement of explained variance in BICDFS when the block of the two fear-related variables was added to the block of the BIPQ variables. In the first step, a forward stepwise linear regression on BICDFS was conducted (entry *p* = 0.05; removal *p* = 0.10), in which the BIPQ items with a *p* < 0.10 in the previous series of linear regressions were used as candidates. The was aimed to build a model with variance reduction and simplicity and mitigate the potential multicollinearity between the BIPQ items. In the second step, a multivariable linear regression model was fitted by entering the significant BIPQ items (*p* < 0.05) in Step-1 model and the two fear-related items as the independent variables. The *R*-square change between the two-step models and the *p*-value of its significance test was reported. The variance inflation factors (VIF) were calculated to check multicollinearity of the final two-step models. All the analyses were performed by the SPSS 24.0 Software (Chicago, Illinois, United States).

## Results

### Distributions of the Studied Variables

The majority of the sample were females (67.3%), aged 36–65 years (56.2%), and married or cohabited with a partner (65.3%). About half of the participants (48.5%) reported their highest education level was secondary school; 70.7% reported no chronic diseases notified by doctors (Table 1).

**Table 1:** Distributions of the studied variables (*N* = 300).

**Variables**	***n (%) /mean ±SD***
*Background characteristics*	
Sex	
Male	98 (32.7)
Female	202 (67.3)
Age (years)	
35 or below	53 (17.8)
36–55	102 (34.3)
56–65	65 (21.9)
above 65	77 (25.9)
Marital status	
Single/divorced/widowed	104 (34.7)
Married/cohabited with a partner	196 (65.3)
Education level	
Primary school or below	53 (17.7)
Secondary school	145 (48.5)
College or above	101 (33.8)
Having chronic disease (e.g., hypertension, diabetes, cancer)	
No	212 (70.7)
Yes	88 (29.3)
*Behavioral intention to consult doctors for flu symptoms (BICDFS)*	
For mild flu symptoms	
Definitely no	81 (27.0)
Probably no	62 (20.7)
Probably yes	72 (24.0)
Definitely yes	85 (28.3)
For severe flu symptoms	
Definitely no	11 (3.7)
Probably no	13 (4.3)
Probably yes	79 (26.3)
Definitely yes	197 (65.7)
The BICDFS Scale	6.08 ± 1.66
*The Brief Illness Perception Questionnaire: if you contracted COVID-19*,	
a. How much would your COVID-19 affect your life? (Consequence)	8.26 ± 1.75
b. How long would you think your COVID-19 would continue? (Timeline)	6.80 ± 2.24
c. How much control would you feel you have over your COVID-19? (Personal control)	5.15 ± 2.20
d. How much would you think your treatment could help your COVID-19? (Treatment control)	6.42 ± 1.75
e. How much would you experience symptoms for your COVID-19? (Identity)	6.95 ± 1.90
f. How concerned would you be about your COVID-19? (Concern)	6.76 ± 2.38
g. How well would you feel you understand your COVID-19? (Understanding)	5.89 ± 1.93
h. How much would your COVID-19 affect you emotionally (e.g., does it make you angry, scared, upset, or depressed)? (Emotions)	5.53 ± 2.50
*Fear of COVID-19 during clinic visits*	
Fear of contracting COVID-19 in clinics if you would visit there to consult doctors in the next weeks	3.37 ± 1.23
Fear of being requested to take up COVID-19 testing if you would visit clinics for flu symptoms in the next weeks	3.04 ± 1.36

*BICDFS, behavioral intention to consult doctors for flu symptoms; COVID-19, coronavirus disease 2019*.

Approximately half of the participants reported he/she would probably (24.0%) or definitely (28.3%) consult doctors if he/she had mild flu symptoms in the next week, while the other half reported they would probably not (20.7%) or definitely not (27.0%) do so. Most of the participants reported they would probably (26.3%) or definitely (65.7%) consult doctors for severe flu symptoms, while a few would probably not (4.3%) or definitely not (3.7%) do so. The *mean score* of the BICDFS scale was 6.08 (*SD* = 1.66).

The *mean score* and *SD* of each BIPQ item was 8.26±1.75 (consequences), 6.80±2.24 (timeline), 5.15±2.20 (personal control), 6.42±1.75 (treatment control), 6.95±1.90 (identity), 6.76±2.38 (concern), 5.89±1.93 (understanding), and 5.53±2.50 (emotions), respectively. The *mean score* and *SD* of the fear of contracting COVID-19 in clinics if visiting there to consult doctors in the next week were 3.37±1.23; that of the fear of being requested to take up COVID-19 testing if visiting clinics for flu symptoms in the next week was 3.04±1.36.

### Associations Between Background Variables and BICDFS

The simple linear regression found the participants who had completed college or above showed a lower level of BICDFS, compared to those with an education level of primary school or below (*standardized b* = −0.21, *p* = 0.01). The education level was thus adjusted in the subsequent regression analysis on BICDFS. Other background variables did not show significant associations with the BICDFS (Table 2).

**Table 2:** Associations between background variables and the behavioral intention to consult doctors for flu symptoms (*N* = 300).

	**Behavioral intention to consult doctors for flu symptoms^a^**	
**Background variables**	***Standardized b***	***p***
Sex		
Male	Reference	–
Female	0.03	0.631
Age (years)		
35 or below	Reference	–
36–55	−0.09	0.259
56–65	0.02	0.825
above 65	0.606	0.545
Marital status		
Single/divorced/widowed	Reference	–
Married/cohabited with a partner	−0.02	0.715
Education level		
Primary school or below	Reference	–
Secondary school	−0.11	0.154
College or above	−0.21	0.01
Having chronic disease (e.g., hypertension, diabetes, cancer)		
No	Reference	–
Yes	0.02	0.689

*COVID-19, coronavirus disease 2019*.

a *Simple linear regression was performed for each background variable*.

### Spearman Correlation Between Variables of Interest

Significant correlations were observed in several pairs of interested variables, such as between the BIPQ dimensions of consequence and concern (ρ = 0.36, *p* < 0.001), and concern and emotions (ρ = 0.56, *p* < 0.001), between emotions and fear of contracting COVID in clinics (ρ = 0.40, *p* < 0.001), and between emotions and the level of BICDFS (ρ = 0.21 *p* = 0.001) (Table 3).

**Table 3:** Spearman correlation between variables of interest (*N* = 300).

**Variables**	**1**	**2**	**3**	**4**	**5**	**6**	**7**	**8**	**9**	**10**
BIPQ (Consequence)	1	–	–	–	–	–	–	–	–	–
BIPQ (Timeline)	0.35^***^	1	–	–	–	–	–	–	–	–
BIPQ (Personal control)	−0.03	0.04	1	–	–	–	–	–	–	–
BIPQ (Treatment control)	−0.10	−0.01	0.16^**^	1	–	–	–	–	–	–
BIPQ (Identity)	0.37^***^	0.28^***^	0.05	0.11	1	–	–	–	–	–
BIPQ (Concern)	0.36^***^	0.37^***^	0.08	−0.14^*^	0.35^***^	1	–	–	–	–
BIPQ (Understanding)	0.10	0.24^**^	0.32^***^	−0.02	0.03	0.19^***^	1	–	–	–
BIPQ (Emotions)	0.28^***^	0.37^***^	0.16^**^	−0.09	0.27^***^	0.56^***^	0.28^***^	1	–	–
Fear of contracting COVID-19 in clinics if you would visit there to consult doctors in the next week	0.21^***^	0.25^***^	0.12^*^	−0.17^**^	0.09	0.33^***^	0.18^**^	0.40^***^	1	–
Fear of being requested to take up COVID-19 testing if you would visit clinics for flu symptoms in the next week	0.17^**^	0.28^***^	0.03	−0.10	0.15^**^	0.22^***^	0.06	0.25^***^	0.51^***^	1
Behavioral intention to consult doctors for flu symptoms	0.14^*^	0.15^**^	−0.02	0.05	−0.02	0.16^**^	0.13^*^	0.21^***^	0.02	0.05

*BIPQ, the brief illness perception questionnaire; COVID-19, coronavirus disease 2019*.

*** *p <0.001*,

** *p <0.01*,

* *p <0.05*.

### Associations Between Interested Independent Variables and BICDFS

After adjusting for the education level, the BIPQ dimensions of consequences (*standardized b* = 0.15, *p* = 0.01), concern (*standardized b* = 0.17, *p* = 0.003), understanding (*standardized b* = 0.21, *p* = 0.001), and emotions (*standardized b* = 0.19, *p* = 0.001) were significantly associated with the level of BICDFS, respectively. The timeline was marginally associated with the level of BICDFS (*standardized b* = 0.11, *p* = 0.052). The five items were hence included in the forward stepwise regression in the hierarchical regression analysis. Both the two fear-related items did not show significant associations with the BICDFS (Table 4).

**Table 4:** Associations between illness representations of COVID-19, fear of COVID-19 during clinic visits, and the behavioral intention to consult doctors for flu symptoms (*N* = 300).

	**Behavioral intention to consult doctors for flu symptoms^a^**	
**Independent variables**	***Standardized b***	***p***
BIPQ (Consequence)	0.15	0.010
BIPQ (Timeline)	0.11	0.052
BIPQ (Personal control)	−0.01	0.843
BIPQ (Treatment control)	0.04	0.480
BIPQ (Identity)	−0.07	0.248
BIPQ (Concern)	0.17	0.003
BIPQ (Understanding)	0.21	0.001
BIPQ (Emotions)	0.19	0.001
Fear of contracting COVID-19 in clinics if you visited there to consult doctors in the next week	0.01	0.815
Fear of being requested to take up COVID-19 testing if you visited clinics for flu symptoms in the next week	0.02	0.695

*BIPQ, the brief illness perception questionnaire; COVID-19, coronavirus disease 2019; N.A., not applicable*.

a *Multivariable linear regression was performed for each independent variable and with the education level being controlled for*.

### Hierarchical Regression on BICDFS

In the step-1 model, understanding (*standardized b* = 0.16, *p* = 0.01) and emotions (*standardized b* = 0.15, *p* = 0.02) exhibited significantly positive associations with the level of BICDFS and were included in the step-2 model. The *R-square* of the step-1 model was 0.079. When adding the two fear-related items to the step-2 model, the significance of understanding (*standardized b* = 0.16, *p* = 0.01) and emotions (*standardized b* = 0.17, *p* = 0.008) remained, and the two fear-related items still did not show significant associations with the BICDFS. The *R-square* change was 0.004, yielding a non-statistically significant change (*p* = 0.49). All the VIFs in the two models were between one and two, which indicated a low level of multicollinearity (Table 5).

**Table 5:** Hierarchical regression on the behavioral intention to consult doctors for flu symptoms (*N* = 300).

	**Intention to consult doctors for flu symptoms**			
	**Step 1^a^**		**Step 2^b^**	
**Independent variables**	***Standardized b***	***p***	***Standardized b***	***p***
BIPQ (Consequence)	N.S.	N.S.	N.A.	N.A.
BIPQ (Timeline)	N.S.	N.S.	N.A.	N.A.
BIPQ (Concern)	N.S.	N.S.	N.A.	N.A.
BIPQ (Understanding)	0.16	0.014	0.16	0.013
BIPQ (Emotional response)	0.15	0.015	0.17	0.008
Fear of contracting COVID-19 in clinics if you would visit there to consult doctors in the next week	N.A.	N.A.	−0.08	0.252
Fear of being requested to take up COVID-19 testing if you would visit clinics for flu symptoms in the next week	N.A.	N.A.	0.02	0.785

*BIPQ, the brief illness perception questionnaire; COVID-19, coronavirus disease 2019; N.S., not selected by stepwise regression; N.A., not applicable*.

a *Step 1: forward stepwise linear regression involving the significant BIPQ items in Table 4, with education level being controlled for*.

b *Step 2: multivariable linear regression by entering the significant BIPQ items in Step 1 and two items of fear of COVID-19 during clinic visits, with education level being controlled for*.

## Discussion

As flu symptoms are clinical features of both COVID-19 and other diseases such as influenza and tuberculosis (9), seeking related medical consultation timely is essential. The study showed that at the time of the study when the second wave of COVID-19 in Hong Kong was just ended, about half of the participants (52.3%) had an intention to consult doctors if they had mild flu symptoms in the next week, and 92.0% presented such an intention if they had severe flu symptoms. According to the health authorities in Hong Kong, all people having respiratory symptoms should seek medical advice promptly during the COVID-19 pandemic (30). The finding implies some Hong Kong residents may not follow the government's suggestion, which may bring health risks to both themselves and the community. No similar studies could be located to compare the result. A 2020 survey showed that about 64.2% of the United States. participants and 79.0% of the United Kingdom. Participants would adopt the recommended care-seeking option (i.e., stay at home and contact the health system) if they recently visited China and had a fever/cough, or had close contact with someone who did (31). The situation was not comparable to the present case.

In our study, people with a higher education level (college or above) presented a lower level of BICDFS. This was unexpected because the education level had been widely found to be positively associated with better care-seeking behaviors, as people with a higher education level usually showed greater awareness of a health problem and thus were more likely to seek appropriate medical help (32–34). Future public advocacy of appropriate health behaviors during the COVID-19 pandemic should pay additional attention to the highly educated group.

The BIPQ dimension of understanding, which refers to the extent to which people perceive they understood the health threat and its implications, was positively associated with a higher level of BICDFS in the hierarchical regression model. It corroborated studies on other diseases (e.g., H1N1, dementia, cancer) which demonstrated that people who scored higher in the dimension of understanding showed better health behaviors or stronger behavioral intentions (35–37). Under the context of COVID-19, those with a comprehensive understanding of COVID-19 may be more positive to cope with it, as they may better realize the necessity and importance of seeking medical consultation for flu symptoms and hence have a higher intention to do it. Also, the dimension of consequences was positively associated with the level of BICDFS in the regression analysis. Understandably, those who perceive the consequences of COVID-19 on his/her life to be more severe may be more triggered to seek prompt medical help for its related symptoms. The finding is hence consistent to some behavioral health theories, such as the Health Belief Model, which postulates positive associations between perceived severity of a health-condition and related positive coping behaviors (38). The association, however, became statistically non-significant in the stepwise regression model, probably due to its correlation with the dimensions of emotions and concern as shown in the correlation analysis. The findings suggest that to promote people's care-seeking behaviors for COVID-19, it is important to increase their comprehensive understanding of the disease and its potential negative impact on everyday life.

The BIPQ dimensions of emotions and concern were positively associated with the level of BICDFS in the regression analyses, respectively; those who had more negative emotions (e.g., becoming angry, scary, upset, or depressed due to COVID-19) and concerns of COVID-19 presented a stronger intention to consult doctors for flu symptoms. Interestingly, it infers that the negative emotions and concerns aroused by COVID-19 may guide people to positively cope with the disease. The direction of the associations was consistent with the previous H1N1 study in Greece (13) but opposite to the H1N1 study in Hong Kong (10). The dimension of concern became non-significantly associated with the BICDFS in the stepwise regression model, probably due to its significant correlation with the dimension of emotions. During the COVID-19 pandemic, it is essential to monitor the negative emotions of the general public and provide advice on the positive coping strategies for the emotions, so as to avoid panic and help prevent and control the disease (13).

Unexpectedly, both the fear of contracting COVID-19 in clinics and the fear of being required to take up COVID-19 testing in clinics did not show significant associations with the BICDFS in the regressions that adjusted and not adjusted for the illness representations. The parallel processes of danger control and fear control of the Extended Parallel Process Model (22) may help explain the non-significant finding: the fear of COVID-19 during clinic visits is highly correlated with the fear emotions aroused by COVID-19; such fear appeal may invoke an action to seek medical consultations for flu symptoms to confirm one's COVID-19 status (a danger control response) and may also lead people to control their fear (a fear control response), instead of control the disease, which results in the avoided medical seeking for COVID-19-related symptoms. The two types of responses may offset each other. People with fear of COVID-19 during clinic visits may be more worried about COVID-19, and thus have a stronger urge to seek medical consultation for flu symptoms to gain peace in mind or to receive early treatment; meanwhile, their care-seeking behaviors for COVID-19 may be deferred by the fear of COVID-19 as a response of fear control. Future studies are warranted to further understand the mechanisms behind the association between fear and care-seeking behaviors during the COVID-19 pandemic.

It is noteworthy that previous literature has shown that levels of medical utilization had decreased during emerging respiratory infectious diseases (e.g., SARS) as HAI was a concern (14, 19). It is important to differentiate the nature of medical consultations involved in different studies. Care-seeking for flu symptoms may be a special case with respect to those involved other illnesses, as COVID-19 and flu share similar symptoms. There is a potential motivation to seek medical consultations and treatment for flu symptoms in the face of having the risk of infecting COVID-19. Negative cognitive/emotional illness representations and related fear may thus be positively associated with the care-seeking behaviors/intentions. For other medical conditions that do not have this feature of similarity, the negative illness representations and fear of COVID-19 may be negatively associated with the related care-seeking behaviors/intentions, as people may choose not to visit health facilities to prevent HAI. Another study conducted by our team based on the same sample population indicated that the prevalence of behavioral intention to visit hospitals for scheduled medical consultations was 62.3% during the COVID-19 pandemic, and fear of HAI was a negatively-associated factor (39). To minimize the concern of HAI, remote medical consultations (e.g., hotline consultation) that can manage diseases and assess the necessity of facility-based services have been suggested during the COVID-19 pandemic (40, 41). Future studies are warranted to investigate and compare levels of care-seeking behaviors for different types of medical consultations during the COVID-19 pandemic and understand the associated factors. Longitudinal research that monitors people's medical care-seeking behaviors during and post the COVID-19 pandemic are also necessary. This is an important research question as people may have to live with COVID-19 for an unforeseeable length of time.

Some limitations should not be ignored. First, this was a cross-sectional study that could not allow us to establish causal relationship. Second, selection bias might exist, because the sample was over-represented by females (67.3%) who only accounted for 54.4% of the Hong Kong population (42) and the response rate was 54.5%. Third, the data were self-reported, which might introduce social desirability bias. Fourth, the measurements for the BICDFS and fear of COVID-19 during clinic visits were derived from previous studies on the SARS and H1N1 outbreaks in Hong Kong and had not been validated in the context of COVID-19 previously. Fifth, other potential factors of BICDFS (e.g., mental health status, history of close contact with COVID-19 cases) were not included in the study. Sixth, the generalization of the findings should be made with caution, as the study was only conducted in a small sample of Hong Kong Chinese people and the participants were recruited within one week at the end of the second wave of COVID-19. The level of BICDFS and its association with illness representations and fears might change if the COVID-19 situation changed. Seventh, the number of people who had actually avoided medical consultations for flu symptoms was unknown, as it would require a large sample to assess. This study was only exploratory; future large-scale studies are required to elaborate this important research question.

## Conclusions

In conclusion, this study found that the Hong Kong general public showed a relatively low level of intention to seek medical consultations for flu symptoms during the COVID-19 pandemic. Such an intention was positively associated with people's comprehensive understanding of COVID-19 and negative emotions aroused by the disease. To promote the general public's care-seeking behaviors for flu symptoms, it may be helpful to raise people's overall understanding of COVID-19 and provide advice on positive coping strategies for emotional arousals.

## Data Availability Statement

The original contributions presented in the study are included in the article/supplementary material, further inquiries can be directed to the corresponding author/s.

## Ethics Statement

The Ethical approval was obtained from the Survey and Behavioral Research Ethics Committee from The Chinese University of Hong Kong (No. SBRE-19-661). Written informed consent for participation was not required for this study in accordance with the National Legislation and the Institutional Requirements.

## Author Contributions

SL and JL: conceptualization. SL, RS, and JL: methodology. SL and ML: formal analysis and investigation. SL: writing—original draft preparation. JL: writing—review and editing. All authors contributed to the article and approved the submitted version.

## Conflict of Interest

The authors declare that the research was conducted in the absence of any commercial or financial relationships that could be construed as a potential conflict of interest.

## Publisher's Note

All claims expressed in this article are solely those of the authors and do not necessarily represent those of their affiliated organizations, or those of the publisher, the editors and the reviewers. Any product that may be evaluated in this article, or claim that may be made by its manufacturer, is not guaranteed or endorsed by the publisher.
